# Medico-Legal Society and Section on Medico-Legal Surgery

**Published:** 1894-11

**Authors:** 


### Medico-Legal Society and Section on Medico-Legal Surgery.

There will be a joint session of the Medico-Legal Society and the Section of Medico-Legal Surgery, at the Academy of Medicine, New York City, on Thursday, November 15th, 1894, at 8 o'clock p. m. precisely.

PROGRAMME.

I.

Session of the Medico-Legal Society, the President, H. W. Mitchell, M. D., in the chair:

*a.* Election of new members and miscellaneous business.

*b.* Nomination of officers for the ensuing year.

II.

Session of the Section on Medico-Legal Surgery, Chief Surgeon, Granville P. Conn, M. D., chairman, in the chair:

*a.* Opening address by Chief Surgeon Granville P. Conn, M. D., on "*Hygienic Training of Men in Charge of Railway Trains.*"

*b.* Discussion of same, limited to five minutes each.

*c.* "*Expert Examination of Plaintiff in Damage Cases, when Ordered by the Court.*" By George Chaffee, M. D., ex-President New York State Society of Railway Surgeons.

*d.* Discussion, limited to five minutes, by Clark Bell, Esq., Judge Roger A. Pryor, Nelson Smith, Esq., Judge Abram H. Dailey, H. W. Mitchell, M. D., Prof. Stillings, of N. H., Chief Surgeon V. C. R. R., M. Cavana, M. D., and ex-Surrogate R. S. Ransom.

*e.* "*The True Line of Duty of the Railway Surgeon.*" By Clark Bell, Esq.

*f.* Discussion, five minutes each, by Surgeon George Chaffee, M. D., Surgeon A. M. Phelps. M. D., R. S. Harnden, M. D., C. M. Daniel, M. D., S. S. Thorne, M. D., J. B. Murdoch, M. D., Nicholas Senn, M. D., W. B. Outten, M. D., G. P. Conn, M. D., and others. Eminent surgeons have been invited to submit views on this subject who can not be present. Reports will be read from various parts of the whole country.

*g.* "*Medical Witnesses.*" By R. S. Harnden, M. D., ex-President Erie Railway Surgeons.

*h.* Discussions, five minutes each, by H. W. Mitchell, M. D., Chief Surgeon Estes, Lehigh Valley R. R., M. Cavana, M. D., C. M. Daniels, M. D., and others.

The New York State Association of Railway Surgeons holds its annual meeting at the Academy of Medicine on same day—morning and afternoon session—to which all our members have been invited, and the members of that Society are cordially invited to attend our meeting, and take part in the discussion.

A general attendance is requested. Members, not on programme, wishing the floor, on either subject, will forward their views, in brief, to the Secretary, if unable to be present. Members will please be prompt in attendance.

For Medico Legal Society,

H. W. MITCHELL, President.
CLARK BELL, Secretary.
F. B. DOWNS, M. D., Asst. Secy.

For Section on Medico-Legal Surgery,

GRANVILLE P. CONN, Chairman.
CLARK BELL, Vice-Chairman and Secy.
GEORGE CHAFFEE, M. D., Treasurer.

